# Differences in the effects of *Bordetella pertussis* and respiratory syncytial virus infection on the composition of nasopharyngeal flora in neonates

**DOI:** 10.3389/fped.2023.1034147

**Published:** 2023-06-07

**Authors:** Yijun Ding, Qing Wang, Dongfang Li, Yue Li, Kaihu Yao, Tianyou Wang

**Affiliations:** ^1^ Department of Neonatology, Beijing Children’s Hospital, Capital Medical University, National Center for Children’s Health, Beijing, China; ^2^ Beijing Pediatric Research Institute, Beijing Children’s Hospital, Capital Medical University, National Center for Children’s Health, Beijing, China; ^3^ R&D Department, BGI PathoGenesis Pharmaceutical Technology, BGI-Shenzhen, Shenzhen, China; ^4^ Department of Hematology and Oncology, Beijing Children’s Hospital, Capital Medical University, National Center for Children’s Health, Beijing, China

**Keywords:** infection, bordetella pertussis, respiratory syncytial virus, neonate, respiratory flora, nasopharynx, diversity

## Abstract

**Introduction:**

*Bordetella pertussis* and respiratory syncytial virus (RSV) are important pathogens causing cough in neonates. Few studies have investigated the differences in the effects of these two specific infections on respiratory flora. The aim of this study was to explore whether infections with *Bordetella pertussis* and RSV have different effects on respiratory floral composition in neonates.

**Methods:**

Nasopharyngeal respiratory flora was assessed by 16S ribosomal RNA amplification and V3–V4 region sequencing. Shannon and Simpson indices were calculated to determine the *α* diversity and principal coordinate analysis was performed to determine the *β* diversity.

**Results:**

In total, 111 hospitalized neonates were divided into the pertussis (*n* = 29), RSV (*n* = 57), and control groups (*n* = 25) according to the pathogens detected. The relative abundance of *Bordetella* was significantly higher in the pertussis group (median: 19.18%, interquartile range: 72.57%). In contrast, this species was not detected in the other two groups. In the RSV group, the relative abundance of *Streptococcus* (median: 77.15%, interquartile range: 45.84%) was significantly higher than those in the pertussis and control groups (both *P *< 0.001). The *α* diversity of the RSV group was significantly lower than that of the control group (*P *< 0.001). Moreover, no statistically significant differences in the Shannon and Simpson indices were observed between the pertussis and control groups (*P *= 0.101 and *P *= 0.202, respectively). Principal coordinate analysis revealed a large overlap between the pertussis and control groups and a significant distance between the RSV and control groups without any overlap.

**Discussion:**

Thus, the effects of infections with the two species, *B. pertussis* and RSV, impacted the diversity of nasopharyngeal flora differently. The principles underlying the difference in the effects of different pathogens on microbial flora require further investigation.

## 1. Introduction

Both *Bordetella pertussis* and respiratory syncytial virus (RSV) can cause respiratory infection in young children. These infections have specific clinical manifestations which are considered during clinical diagnosis. Although the vaccination coverage for *B. pertussis* infection is high, it is still a public health issue that seriously threatens infant health (1). Acute lower respiratory tract infection caused by RSV is also a major cause of hospitalization in children under 5 years of age (2).

The nasopharyngeal tract is home to a special ecosystem that may change during infection and plays a key role in the host's susceptibility to pathogens and the occurrence and development of diseases. Previous studies have shown that RSV-induced acute lower respiratory tract infection in early life is closely related to the development of asthma in children (3). RSV may induce changes in the composition of the nasopharyngeal microflora, thereby increasing the risk of asthma (4). However, recent studies have shown that RSV infection did not increase the risk of early asthma by 2 years of age (5). Therefore, the impact of RSV on the nasopharyngeal microbiota and later diseases is still uncertain. Some mouse models have been used to reveal that the microbiota residing in the nasopharynx prevent the colonization of *B. pertussis* (6), but the characteristics of changes in the nasopharyngeal flora of humans after infection with *B. pertussis* are still unclear. Overall, few studies compare the differences between the infections in terms of the changes in the nasopharyngeal flora of neonates. Therefore, the aim of this study was to compare the nasopharyngeal floral composition between hospitalized neonates with and without pertussis and RSV infection. Understanding how nasopharyngeal ecology is regulated after infection by these pathogens may help to understand the determinants of the disease and its potential diagnosis, prevention, and treatment.

## 2. Materials and methods

### 2.1. Study design

The neonates hospitalized in the neonatal center of Beijing Children's Hospital from November 20, 2015 to August 6, 2019 were included in this study. Their gestational age was ≥34 weeks, age ≤28 days, and birth weight ≥2,000 g. Based on the clinical diagnosis and pathogens detected, the neonates were divided into a *B. pertussis*-positive group and RSV-positive group. The diagnostic criteria for pertussis includes observation of the specific clinical manifestations and a positive *Bordetella* pertussis culture or positive quantitative real time polymerase chain reaction (qPCR) test results for pertussis (7). RSV infection was confirmed using direct immunofluorescence assay. The control group included neonates without infectious diseases, who were hospitalized due to pathological jaundice, and had not been administered antibiotics prior to admission. Neonates with underlying medical conditions, such as congenital heart disease, chronic pulmonary disease, and immunodeficiency were excluded.

This study was conducted in accordance with the tenets of the Declaration of Helsinki and approved by the Ethics Committee of Beijing Children's Hospital. Written informed consent was obtained from the parents of all participants.

### 2.2. Detection of *B. pertussis*

Two nasopharyngeal swabs were collected from all neonates who met the selection criteria, in accordance with standard protocol (8). One nasopharyngeal swab was immediately marked with a “Z” at the bedside and used to inoculate carbon agar plates (Oxoid, Hampshire, United Kingdom) containing 10% defibrinated sheep blood and *Bordetella* selective supplement (cephalexin). The plates were then incubated in a humidified incubator at 35°C for a maximum of 7 days and checked daily for bacterial growth on days 3–7. After incubation, potential colonies were confirmed by slide agglutination test (Remel Europe Ltd., Dartford, UK). The sample with positive agglutination reaction contains *B. pertussis*, while the sample with negative agglutination reaction is *B. parapertussis*. The other nasopharyngeal swab was immediately placed in aseptic cryopreservation tubes and stored at −80°C. DNA extraction, qPCR with the extracted DNA, and 16sRNA sequencing were performed within 6 months.

The DNA was extracted using an extraction kit (SBS Genetic Co., Ltd., Beijing, China) following the manufacturer's instructions. The extracted DNA was stored at −20°C for further analysis. qPCR was performed using the pertussis nucleic acid detection kit (DaAn Co. Ltd., Guangzhou, China). A crossing threshold (Ct) value ≤38 was judged as positive. The specific process is the same process followed in our previous research (9).

### 2.3. RSV detection

Sputum specimens were collected from neonates with lower respiratory tract infections, including bronchitis and pneumonia, for RSV testing on the day of admission. RSV was detected using direct immunofluorescence assay according to the manufacturer's instructions. If the neonate tested positive for RSV, a nasopharyngeal swab sample was collected from them immediately.

### 2.4. 16S rRNA gene sequencing and compositional analysis

#### 2.4.1. DNA extraction and PCR amplification

Microbial DNA was extracted from nasopharyngeal swab samples using the E.Z.N.A. soil DNA Kit (Omega Bio-tek, Norcross, GA, USA) according to the manufacturer's protocol. The 16S rRNA V3–V4 region was amplified by qPCR using the 338F/806R primers (primer sequence, F: 5′-ACTCCTACGGGAGGCAGCAG-3′; R: 5′-GGACTACHVGGGTWTCTAAT-3′) (10). qPCR was carried out on an ABI GeneAmp 9,700 system (Applied Biosystems, Waltham, MA, USA) in a 20 µl amplification system consisting of 4 µl of 5 × FastPfu buffer, 2 µl of dNTPs (2.5 mM), 0.8 µl of forward primer (5 mM), 0.8 µl of reverse primer (5 mM), 0.4 of µl FastPfu polymerase, 0.2 µl of bovine serum albumin, and 10 ng of DNA template; ddH_2_O was added to make up the volume to 20 µl. The cycling conditions for the PCR reaction were as follows: pre-denaturation at 95°C for 3 min; 27 cycles of denaturation at 95°C for 30 s, annealing at 55°C for 30 s, and elongation at 72°C for 45 s; and final extension at 72°C for 10 min. The PCR products were recovered using a 2% agarose gel and purified using an AxyPrep DNA Gel Extraction Kit (Axygen Biosciences, Union City, CA, USA). DNA quantification was carried out using a QuantiFluor-ST system (Promega, Madison, WI, USA) according to the manufacturer's instructions.

#### 2.4.2. Illumina MiSeq sequencing

As per Majorbio Bio-Pharm Technology Co. Ltd. (Shanghai, China) standard procedures, the purified amplicons were combined in equimolar amounts and paired-end sequenced (2  ×  300 bp) on the Illumina MiSeq platform (Illumina, San Diego, CA, USA).

#### 2.4.3. Sequencing data processing

Raw data were quality-filtered using Trimmomatic and merged using FLASH according to the following method (11): (1) the reads were removed at any site with an average quality score <20 over a 50 bp sliding window; (2) sequences with an overlap length of more than 10 bp were merged, with mismatch not exceeding 2 bp; and (3) sequences of each sample were separated according to barcodes (exactly matching) and primers (allowing 2 nucleotide mismatches), and reads containing ambiguous bases were removed. Paired-end reads with >10 base overlaps and <2 mismatches were selected for the assembly of high-quality tags. Using UPARSE (Version 7.1, http://drive5.com/uparse/), the assembled tags were clustered using operational taxonomic units (OTUs), and the similarity cut-off value was set at 97%. Clustering was used to remove single sequences and inlays. To ensure the accuracy of the results obtained, we removed OTUs with abundance values less than 0.001% of the total number of reads. The taxonomy of each 16S rRNA gene sequence was verified using the RDP classifier algorithm (http://rdp.cme.msu.edu/) against the Silva (SSU123) 16S rRNA database, at a confidence threshold of 70%. To strengthen quality control and avoid contamination during the operation process, we used the same approach for DNA extraction, PCR amplification, and 16S rRNA sequencing on clean nasopharyngeal swabs, and used aseptic cryopreservation tubes as negative controls.

### 2.5. Statistical analysis

SPSS statistics version 23.0 (IBM Corp., Armonk, NY, USA) and R version 3.4.0 (Foundation for Statistical Computing, Vienna, Austria) were used for statistical analyses. Continuous variables are represented as median [interquartile range (IQR)] or mean ± standard deviation (SD), and categorical variables are represented as frequencies and percentages. The chi-square test and the Wilcoxon rank-sum test were used to evaluate the differences between the groups. Kruskal–Wallis test was used to compare the differences among the three groups. Bonferroni-corrected Mann–Whitney *U*-test was used for pairwise comparison. The Shannon and Simpson indices were calculated to elucidate the *α* diversity. Kruskal–Wallis test was used to compare the *α* diversity of samples among the three groups. Similarity matrix between the samples in each group were based on the Bray–Curtis algorithm (Vegan function in R). Principal coordinate analysis (PCoA) was used to elucidate the *β* diversity of the samples (Vegan function in R). Two-tailed *P*-values < 0.05 were considered statistically significant.

## 3. Results

### 3.1. Demographic characteristics of the study participants

A total of 29 pertussis-positive, 57 RSV-positive, and 25 control group neonates were included in this study. The baseline characteristics of the study participants are presented in Table 1. Except for the time of sample collection, no significant difference in basic clinical information was observed among the three groups (Table 1). The median sampling time in the RSV group was slightly longer than that in the other groups, mainly because the neonates in this group were tested for RSV first.

**Table 1:** Demographics and clinical parameters of hospitalized neonates in the pertussis, RSV, and control groups.

	Pertussis group	RSV group	Control group	*P*
	(*n* = 29)	(*n* = 57)	(*n* = 25)	
Age (days)	20.1 ± 5.8	18 (13, 25)	17.5 ± 5.6	0.277
Male sex	14 (48.3%)	34 (59.6%)	10 (40.0%)	0.230
Gestational age (weeks)	39 [1]	39 [2]	38 [2]	0.041
Birth weight (g)	3,330 [410]	3,351.5 ± 465.2	3,302.8 ± 482.6	0.754
Birth by cesarean section	15 (51.7%)	21 (36.8%)	11 (44.0%)	0.411
Breastfeeding	22 (75.9%)	48 (84.2%)	18 (72.0%)	0.395
Duration of hospital stay at sampling (days)	1 [3]	3 [2]	1.0 [1]	<0.001
Numbers of neonates exposed to antibiotics before sampling	29 (100%)	57 (100%)	0 (0%)	<0.001
Use time of antibiotics as of sampling (days)	2 [5]	4 [3]	/	0.065

Continuous variables that conform to a normal distribution are expressed as the mean ± standard deviation. Those that are not normally distributed are expressed as the median [interquartile range]. Categorical variables are expressed as the number of cases (percentage). *P *< 0.05 indicates a statistically significant difference.

### 3.2. Operational taxonomic unit cluster analysis

Twenty-nine samples were obtained from the *B. pertussis* group, and 38,586 high-quality sequence tags on average were obtained for each sample (range: 28,207–52,292 tags/sample, median: 40,178 tags/sample, IQR: 9,253 tags/sample). The median of the OTUs was 84/sample (IQR: 167). A total of 57 samples were collected from the RSV group, and 45,161 high-quality tags were obtained from each sample (range: 31,124–79,645 tags/sample, median: 43,244 tags/sample, IQR: 10,482 tags/sample). The median of the OTUs was 158 OTUs for each sample (IQR: 110 OTUs/sample). A total of 25 samples were collected from the control group, 43,595 high-quality tags were obtained for each sample on average (range: 29,999–56,059 tags/sample, median: 42,689 tags/sample, IQR: 10,979 tags/sample), and the median was 61 OTUs/sample (IQR: 35 OTUs/sample). At the phylum level, the three groups were dominated by *Firmicutes*, *Proteobacteria*, and *Actinobacteria*, accounting for more than 90% of all samples. *Staphylococcus*, *Streptococcus*, *Bordetella*, *Corynebacterium*, *Pseudomonas*, *Ralstonia*, and *Enterobacter* were the seven major genera that contributed more than 80% in terms of the relative abundance of the microbes.

### 3.3. Differences in the microflora composition among the pertussis, RSV, and control groups

**Figure 1:**
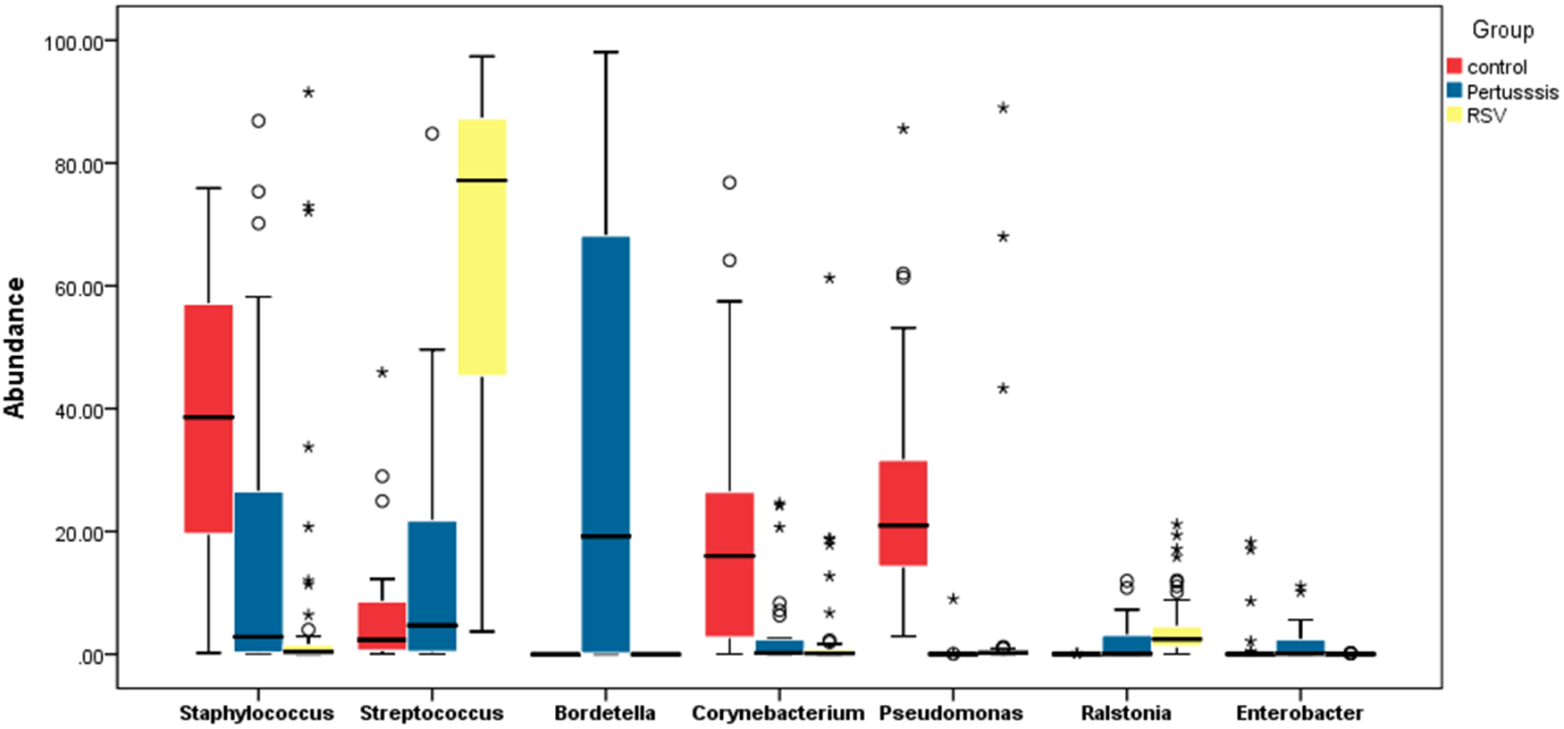
Comparative boxplot of the relative abundance of the major genera found in the nasopharynges of hospitalized neonates. The 16S rRNA sequencing method was used to determine the composition of the nasopharyngeal flora. The abscissa represents the 7 major bacterial genera in the study participants. The ordinate represents the percentage relative abundance of the genera. The Wilcoxon rank-sum test was used to assess the statistical significance of the differences between the relative abundance of the major bacterial genera in neonates among the pertussis, RSV, and control groups. ***P < 0.001, **P < 0.01, *P < 0.05.

At the genus level, the relative abundances of the seven dominant bacterial genera in the pertussis, RSV, and control groups were significantly different except for *Enterobacter* (Table 2). The relative abundance of *Bordetella* was the highest in the pertussis group (median: 19.18%, IQR: 72.57%), whereas *Bordetella* was not detected in both RSV and control group. In the RSV group, the relative abundance of *Streptococcus* was the highest (median: 77.15%, IQR: 45.84%), which was statistically significant compared with the control group (all *P *< 0.001). However, the relative abundance of *Streptococcus* was not significantly different between the pertussis and control groups (*P *= 0.381). The control group was dominated by *Staphylococcus* in terms of relative abundance, which was significantly higher than in the pertussis and RSV groups (*P *= 0.006 and *P *< 0.001, respectively). Compared with the RSV group, the relative abundance of *Staphylococcus* was also higher in the pertussis group (*P *= 0.009) (Figure 1).

**Table 2:** Comparison of relative abundance of dominant bacteria in the pertussis, RSV, and control groups.

Bacteria	Pertussis group (*n* = 29)	RSV group (*n* = 57)	Control group (*n* = 25)	*P*
	Median (IQR)			
Staphylococcus	2.84% (35.83%)	0.39% (1.55%)	38.61% (39.22%)	<0.001
Streptococcus	4.67% (24.79%)	77.15% (45.84%)	2.35% (8.39%)	<0.001
Bordetella	19.18% (72.57%)	0	0	<0.001
Corynebacterium	0.22% (2.42%)	0.13% (0.84)	16% (25.06%)	<0.001
Pseudomonas	0.01% (0.02%)	0.25% (0.31%)	20.96% (22.11%)	<0.001
Ralstonia	0.07% (3.28%)	2.45% (3.53%)	0.01% (0.02%)	<0.001
Enterobacter	0.1% (2.91%)	0.02% (0.05%)	0.03% (0.23%)	0.083

### 3.4. Microbial diversity

#### 3.4.1. *α* diversity

**Figure 2:**
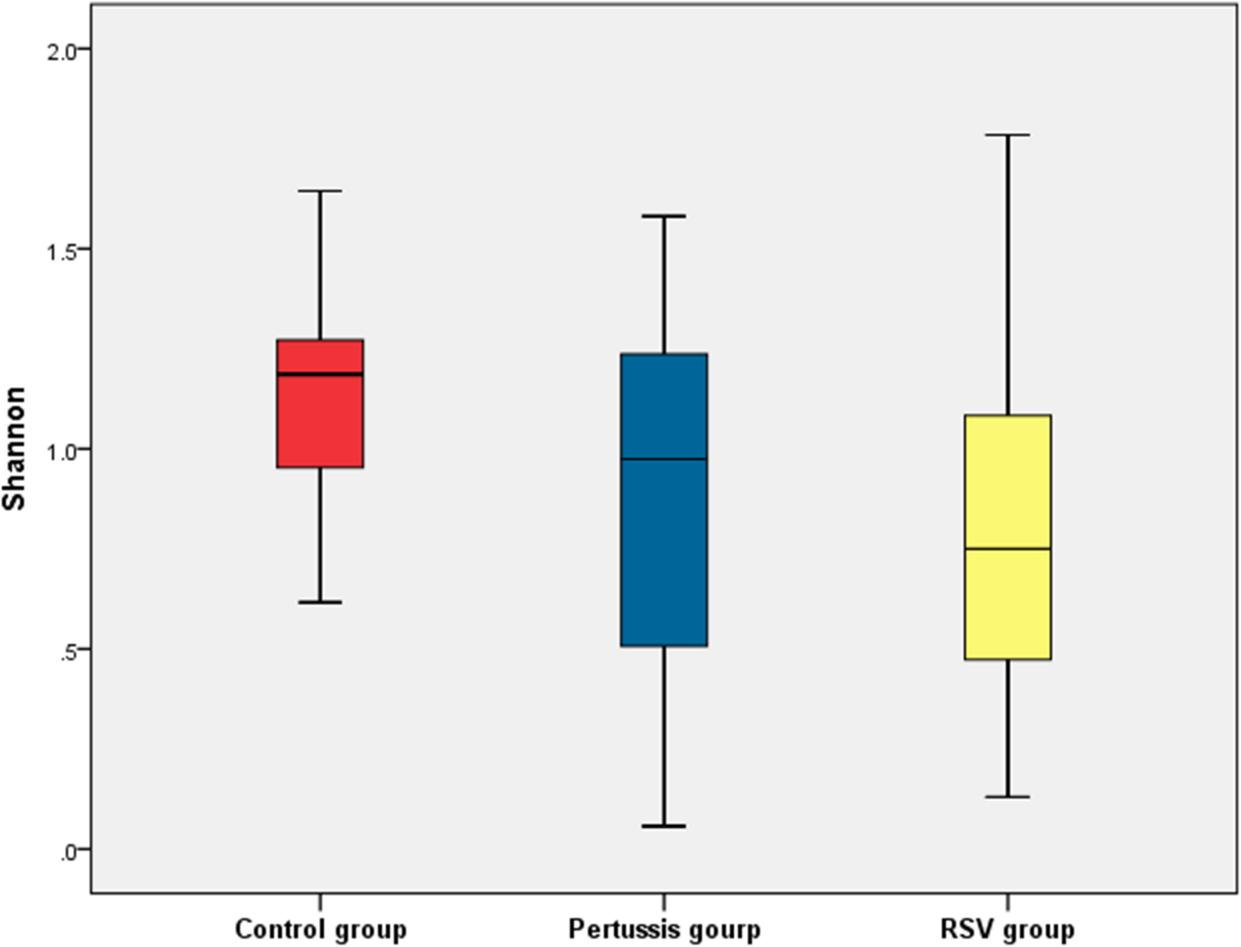
Shannon index of *α* diversity in the control, pertussis and RSV groups.

**Figure 3:**
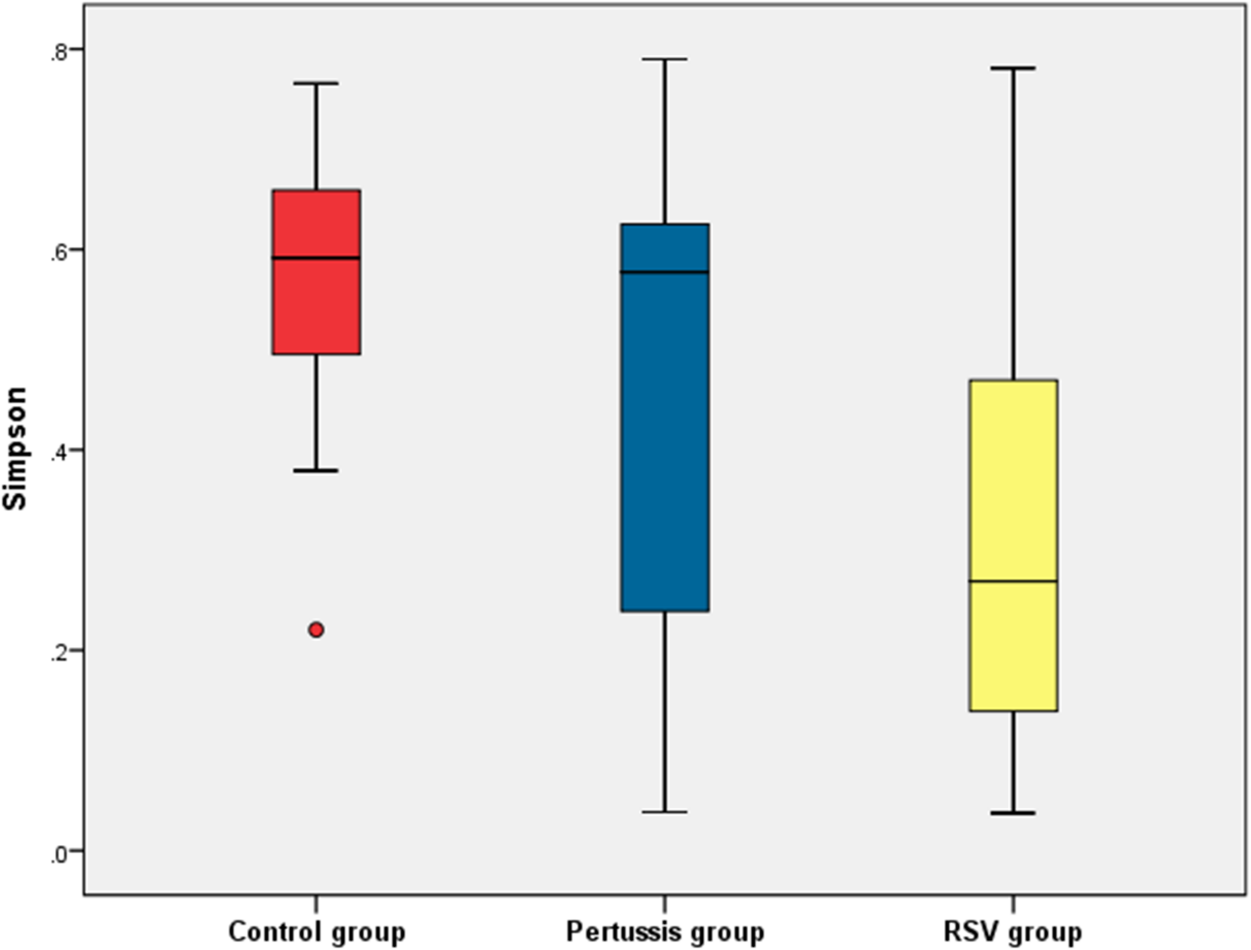
Simpson index of *α* diversity in the control, pertussis and RSV groups.

At the genus level, statistically significant differences in the mean values of both the Shannon and Simpson indices were observed between the pertussis, RSV, and control groups (*P *= 0.005 and *P *< 0.001, respectively). It is suggested that the *α* diversity of samples among the three groups was significantly different. However, no statistically significant differences in the Shannon and Simpson indices of the pertussis group compared with the control group (*P *= 0.101 and *P *= 0.202, respectively) were noted. The Shannon index of the pertussis group and the control group was 0.89 ± 0.50 and 1.14 ± 0.28, respectively, whereas the Simpson index was 0.45 ± 0.25 and 0.56 ± 0.14, respectively (Figures 2, 3), indicating no significant difference in the *α* diversity between the pertussis and control groups.

In contrast, the Shannon index in the RSV group was significantly lower than that in the control group (0.82 ± 0.41 vs. 1.14 ± 0.26, *P *< 0.001,). The Simpson index in the RSV group was also significantly lower than that in the control group (0.31 ± 0.20 vs. 0.56 ± 0.14, *P *< 0.001), suggesting that the *α* diversity was significantly lower in the RSV group than in the control group (Figures 2, 3).

There was no significant difference in the Shannon index between the pertussis and RSV groups (*P *= 0.354). However, a statistically significant difference in the Simpson index was observed between the two groups (*P *= 0.016) (Figures 2, 3). This suggests the existence of a certain difference in *α*-diversity between the RSV and pertussis groups that is not as large as that between the RSV and control groups.

#### 3.4.2. *β*-diversity

**Figure 4:**
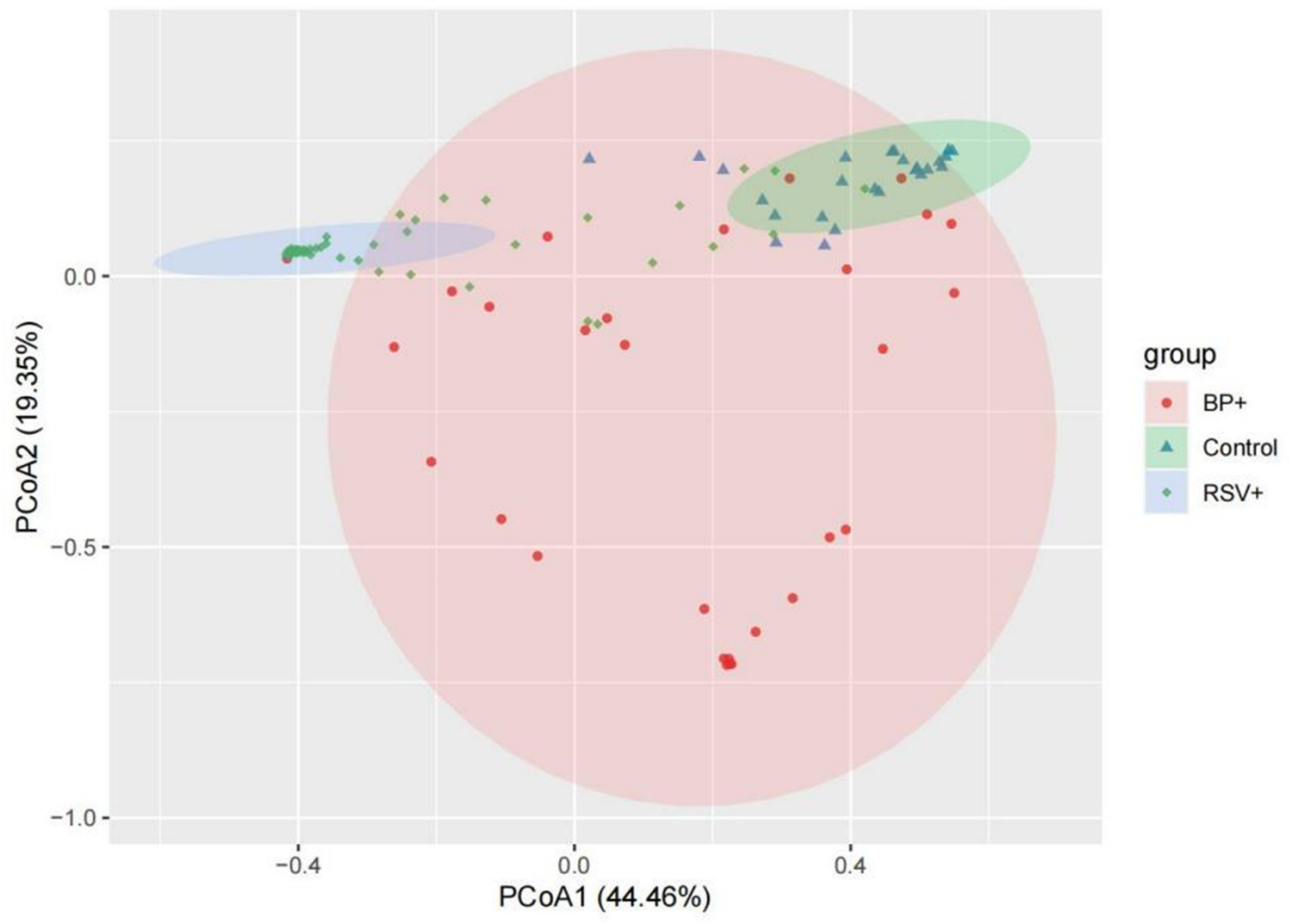
Principal coordinate analysis (PCoA) of *β* diversity in the pertussis, RSV, and control groups. Pink, green, and blue represent the pertussis, control, and RSV groups, respectively. The distance between the two points represents the difference in microbial composition between samples. The large overlap of the two groups of microbiotas suggests that there was little difference in the composition of the microbiota between the two groups of samples. The greater the distance between the two points, the greater the difference in the bacterial community structure between the two groups.

The dissimilarity between the samples in each group based on the Bray-Curtis algorithm. PERMANOVA *P *< 0.05 indicating that the structure of the bacterial community among the three groups was significantly different. PCoA, which was used to evaluate the *β* diversity, revealed that the distance among the pertussis, RSV, and control groups. The PCoA map showed that the control group mostly overlapped with the pertussis group, suggesting that there was little difference in the composition of the flora between these two groups (Figure 4). In contrast, the distance between the RSV group and the control group was large, resulting in no overlap, indicating a significant difference in floral structure between the RSV and control groups (Figure 4). A small overlap between the RSV and pertussis group samples was noted, which indicated a certain difference in the structure of bacterial flora between the two groups that was not as large as the difference between the RSV and control groups.

## 4. Discussion

According to the results obtained in the present study, compared with the control group, there were no significant changes in the *α* diversity of the nasopharyngeal microflora of neonates infected with *B. pertussis*. This finding is consistent with the results of previous animal studies, which have shown that *B. pertussis* cannot replace other microorganisms and occupy the entire nasal cavity of mice after inoculation for 3 days as abundantly as *B. bronchiseptica*. *Bordetella pertussis* requires larger doses to colonize murine nasal cavities and does not displace host microorganisms (6). The specific mechanism behind this characteristic is not clear, but it is thought that the lack of factors necessary for the competition of *B. pertussis* with the microbiota of the nasopharynx may be responsible. The colistin, phage, and CDI systems have been found to be essential factors for the participation of *E. coli* in bacterial competition (12–14). In *Pseudomonas*, *Vibrio*, and *Serratia*, the type VI secretion system (T6SS) is required for microbial competition (15–17). *Bordetella bronchiseptica* competes with other bacterial groups in the murine nasal cavity and is related to the T6SS locus, which is missing in *B. pertussis* (18).

Unlike the *B. pertussis*-infected neonates, the RSV-infected neonates showed lower nasopharyngeal floral *α* diversity than control neonates did. Rosas-Salazar et al. found that the taxonomic composition of the healthy and RSV-infected nasopharyngeal microbiome differed at both the genus and OTU levels, with a marked decrease in the richness of the nasopharyngeal microbiome during acute RSV infection (4). Previous studies have shown that a decrease in nasopharyngeal floral diversity increases the frequency of upper respiratory tract infections (19). In addition, Rosas-Salazar et al. found that nasopharyngeal flora was dominated by *Moraxella*, *Haemophilus*, and *Streptococcus* in RSV-infected infants with a median age of 22 weeks (4). De Steenhuijsen Piters et al. (20) observed that the nasopharyngeal microflora has an effect on disease severity and the abundance of *H. influenzae* and *Streptococcus* strains is positively correlated with hospitalization rates due to RSV infection. Similarly, the present study also found that the relative abundance of nasopharyngeal *Streptococcus* was significantly increased in RSV-infected hospitalized neonates. An abnormally high relative abundance of *Streptococcus* in the nasopharynx is not a good sign. Previous studies have shown that *Streptococcus* strain-dominated profiles in early life predict an unstable floral pattern, are associated with an increased risk of developing pneumonia and bronchitis (21, 22), and are risk factors for future asthma development (23). This may explain, from a micro-ecological perspective, why infants who develop RSV infection early in life are prone to developing recurrent respiratory tract infections and asthma later in life.

Tozzi et al. (24) found that when infection is triggered, the presence of infectious agents may further change the nasal microenvironment, which is contributing to the overgrowth of other commensal bacteria that change into pathobionts, thus affecting the severity of the disease. Therefore, it is important to understand the impact of different pathogenic infections on nasal microbiota ecological. Our findings enable a better understanding of the nasopharyngeal microecology of hospitalized neonates infected with pertussis and RSV and reveal the distinct difference in microecological changes between these two specific infections. However, this study has certain limitations. First, we were unable to analyze bacterial diversity beyond the genus level. Second, both the pertussis group and RSV group used antibiotics before sample collection. The application of antibiotics may have an impact on the flora of the nasopharynx, but our study is based on the analysis of changes in the flora of neonates with pertussis and RSV infection under the rational use of antibiotics. Third, our control group consisted of hospitalized neonates with neonatal jaundice but without infectious disease. It is unclear whether jaundice affects the composition of the nasopharyngeal microecology or abundance of bacterial genera. Nonetheless, the composition of the nasopharyngeal flora in our control group was similar to that in healthy infants, as previously reported (22). Fourth, this study was cross-sectional and had a small sample size. Fifth, we did not control for potential confounding factors. As the study subjects were neonates, we did not consider smoking as a risk factor that may affect the nasopharyngeal flora; however, we could not confirm exposure to secondhand smoke. Further large-scale, longitudinal studies that will control for potential confounding factors are needed to better elucidate changes occurring in the nasopharyngeal flora in infants infected with *B. pertussis* and RSV.

## 5. Conclusions

We found significant differences in the composition of the nasopharyngeal microflora between neonates with pertussis and those with RSV infection. The related mechanism of the differential effects of different pathogens on the nasopharyngeal microbiota must be studied further.

## Data Availability

The datasets presented in this study can be found in online repositories. The names of the repository/repositories and accession number(s) can be found below: https://www.ncbi.nlm.nih.gov/genbank/, PRJNA599511.
